# Cyclodextrin-Based Nanohydrogels Containing Polyamidoamine Units: A New Dexamethasone Delivery System for Inflammatory Diseases

**DOI:** 10.3390/gels3020022

**Published:** 2017-06-08

**Authors:** Monica Argenziano, Chiara Dianzani, Benedetta Ferrara, Shankar Swaminathan, Amedea Manfredi, Elisabetta Ranucci, Roberta Cavalli, Paolo Ferruti

**Affiliations:** 1 Department of Drug Science and Technology, University of Torino, via P. Giuria 9, 10125 Torino, Italy; monica.argenziano@unito.it (M.A.); chiara.dianzani@unito.it (C.D.); benedetta.ferrara@unito.it (B.F.); 2 Department of Ophthalmology, University of Tennessee Health Science Center, Memphis, TN 38163, USA; sswamin4@uthsc.edu; 3 Dipartimento di Chimica, Università degli Studi di Milano, via C. Golgi 19, 20133 Milano, Italy; amedea.manfredi@unimi.it (A.M.); elisabetta.ranucci@unimi.it (E.R.); paolo.ferruti@unimi.it (P.F.)

**Keywords:** dexamethasone, cyclodextrin/polyamidoamine nanohydrogels, topical delivery, β- and γ-Cyclodextrins, COX-2 expression

## Abstract

Glucocorticoids are widely prescribed in treatment of rheumatoid arthritis, asthma, systemic lupus erythematosus, lymphoid neoplasia, skin and eye inflammations. However, well-documented adverse effects offset their therapeutic advantages. In this work, novel nano-hydrogels for the sustained delivery of dexamethasone were designed to increase both bioavailability and duration of the administered drug and reducing the therapeutic dose. Hydrogels are soft materials consisting of water-swollen cross-linked polymers to which the insertion of cyclodextrin (CD) moieties adds hydrophobic drug-complexing sites. Polyamidoamines (PAAs) are biocompatible and biodegradable polymers apt to create CD moieties in hydrogels. In this work, β or γ-CD/PAA nanogels have been developed. In vitro studies showed that a pretreatment for 24–48 h with dexamethasone-loaded, β-CD/PAA nanogel (nanodexa) inhibits adhesion of Jurkat cells to human umbilical vein endothelial cells (HUVEC) in conditions mimicking inflammation. This inhibitory effect was faster and higher than that displayed by free dexamethasone. Moreover, nanodexa inhibited COX-2 expression induced by PMA+A23187 in Jurkat cells after 24–48 h incubation in the 10^−8^–10^−5^ M concentration range, while dexamethasone was effective only at 10^−5^ M after 48 h treatment. Hence, the novel nanogel-dexamethasone formulation combines faster action with lower doses, suggesting the potential for being more manageable than the free drug, reducing its adverse side effects.

## 1. Introduction

Glucocorticoids (GCs) are anti-inflammatory and immunosuppressive agents widely used to treat systemic autoimmune diseases and a number of different conditions, such as asthma, skin diseases, eyes inflammations, allergic reactions, and cancers. They are also common medications in palliative care [1,2]. Despite their clinical efficacy, GCs cause several adverse reactions limiting their clinical usefulness. These adverse effects are mostly dependent on the duration and dosage of the therapy and are, therefore, particularly relevant in chronic diseases that require long-lasting treatments [3,4].

Dexamethasone is a potent glucocorticoid with a high effectiveness in downregulating the expression of anti-inflammatory cytokines. It is used to treat many ocular diseases and it is mainly administered as eye-drops. Unfortunately, high drug concentrations can lead to severe local and systemic side-effects. Moreover, eye-drops are rapidly cleared and consequently frequent instillations of high doses of dexamethasone are required [5].

To enhance the efficacy of dexamethasone and control the release kinetics to the target site in the meantime, decreasing doses and minimizing toxicity, efficient biodegradable and biocompatible nanodelivery systems have been studied. Nanoparticles present a number of advantages for the ocular delivery of drugs, comprising controlled drug release, drug targeting, increased surface adhesion, and drug penetration through mucus membranes. Various nanocarriers with different characteristics and architectures have been studied for the topical delivery of dexamethasone. A number of liposomes have been proposed as dexamethasone delivery systems. The effects of formulation parameters on the liposome physico-chemical properties were deeply investigated, showing that dexamethasone incorporation and release from liposomes was dependent on the type of lipid used and their sizes [6,7]. Previously, β-cyclodextrin-based nanosponges were developed for dexamethasone ocular delivery. Nanosponge formulations showed prolonged drug release kinetics and an increase of dexamethasone corneal permeability compared to marketed formulations [8]. Moreover, “smart” polymer nanoparticles responsive to external stimuli, such as temperature and pH values, or with mucoadhesion properties, received much attention to improve the therapeutic effects and minimize side effects. Polymer nanoparticles able to swell with the pH values can be exploited for the design of nanoformulations with controlled and targeted release kinetics suitable for ocular administration.

Very recently, dexamethasone-loaded Eudragit^®^ RS (an anionic acrylate polymer) and ethyl cellulose nanoparticles exhibited the capability to adhere to the corneal surface and release the drug slowly and in a controlled manner over time [9]. Particularly, dexamethasone showed slower release kinetics in pH 4.5 acetic buffer than in pH 7.5 phosphate buffer. These results are in line with the swelling behavior of the acrylate polymer.

In addition, pH-sensitive polymeric nanoparticles showed a great potential for dermal and transfollicular dexamethasone delivery [10].

In this context, recent advances in drug nanotechnology underline the role played by nanogels for the delivery of dexamethasone [11]. Nanogels are nanostructures consisting of hydrophilic polymer networks and are promising vehicles for the delivery of a variety of different therapeutic agents [12]. Swelling and shrinkage of nanogels can be induced by changes in pH or temperature providing a triggered drug release [13]. Nanogels of methylcellulose hydrophobized with *N*-tert-butylacrylamide and containing dexamethasone were formulated in order to improve the topical ocular therapy by reducing the dosage and frequency of administration. Interestingly, cyclodextrins (CDs) could be integrated as functional units of nanogels in polymer networks acting as carriers of molecules with poor water solubility. Moreover, CD-based nanogels provide useful functionalities, such as effective bioconjugation, good adhesion to surfaces, controlled drug complexation, and drug release [14]. Moya-Ortega and colleagues showed that dexamethasone γ-cyclodextrin-based nanogel eye drops increased ocular bioavailability and gave high drug concentrations in the aqueous humor for at least 3 h after ocular administration in rabbits [15].

Polyamidoamines (PAAs) are biocompatible and biodegradable synthetic polymers apt to create hydrogels, and previously exploited for nanomedicine formulations [16].

Combining PAAs and cyclodextrins, a new nanogel platform was designed aimed at improving the topical administration of dexamethasone.

Here, we report on the development and in vitro characterization of a new ocular nanoformulation of dexamethasone, i.e., dexamethasone nanogel, attempting to ameliorate its therapeutic index by increasing the efficacy and decreasing the side effects.

## 2. Results

### 2.1. Characterization of Blank and Dexamethasone-Loaded Nanogels

The synthetic procedure tuned for preparing cyclodextrin-based nanogels allowed to obtain either β- or γ-cyclodextrin moieties in the hydrogel network. Two hydrogels were prepared as previously described [17] with modifications and were named β-CD/PAA and γ-CD/PAA, respectively.

**Figure 1:**
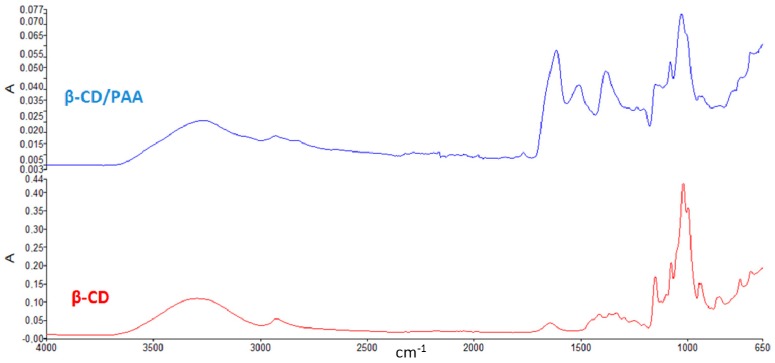
FTIR spectra of β-CD and β-CD/PAA.

The Fourier transformed infrared (FTIR) spectra of β-CD/PAA compared to β-CD are reported in Figure 1.

The presence of peaks around 1500 cm^−1^ detectable only in the spectrum of β-CD/PAA confirmed the interaction of β-CD with the cross-linked units.

The two hydrogels were then reduced to nanometric size by the High Pressure Homogenization (HPH) technique, a top-down technology currently used as a pharmaceutical process for producing colloidal systems [18]. The procedure consisted of forcing a coarse hydrogel suspension in water with a piston having an applied pressure between 100 and 1500 bar through a tiny gap (5–10 μm). The mechanical stresses caused fragmentation of hydrogel matrices, thus producing nanosized particles with the same swelling capability of the parent hydrogels. Interestingly, β-CD/PAA and γ-CD/PAA nanohydrogels were pH sensitive due to the amphoteric nature of their PAA portion. In particular, the swelling degree of both types of nanogels increased regularly from pH 4.0 to 7.4.

**Figure 2:**
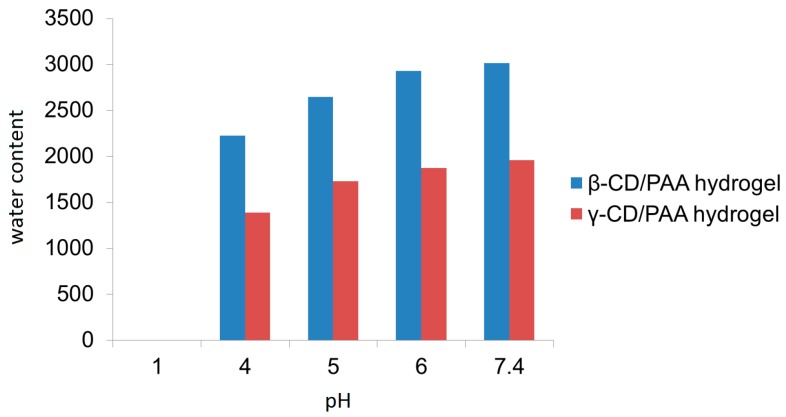
pH Dependence of β-CD/PAA and γ-CD/PAA nanogel swelling in aqueous media.

Figure 2 reports the swelling degree of the two nanogels as a function of the pH value of the external medium. It may be observed that β-CD/PAA nanogel always exhibited a higher water uptake capability than γ-CD/PAA.

The β-CD/PAA nanogel showed a higher swelling degree compared to γ-CD/PAA nanogel; in particular at pH 7.4 an increase of 46% on the nanogel water uptake capability was observed.

Both unloaded nanogels showed average sizes of about 300 nm with a narrow size distribution and negative surface charge. The incorporation of dexamethasone was easily achieved without organic solvents. β-CD/PAA and γ-CD/PAA nanogels were able to incorporate dexamethasone with a loading capacity of 5.15% and 3.61%, respectively.

The physico-chemical characteristics of dexamethasone-loaded β-CD/PAA and γ-CD/PAA nanogels are reported in Table 1. Dynamic light scattering (DLS) analyses showed average diameters of about 310 nm and 370 nm for dexamethasone-loaded β-CD/PAA and γ-CD/PAA nanogels, respectively. The low polydispersity indices indicated a rather uniform population distribution due to the HPH step performed during the nanogel preparation. The zeta potential determination evidenced highly negative surface charges, with a ζ potential of about −30 mV, values essential for the physical stability of nanosuspensions, the electrostatic repulsions avoiding nanogel aggregation. Moreover, the negative charge can favor the interaction with mucin, which is positively charged. Indeed, a large amount of mucin was absorbed on the two nanogel surfaces, reaching a mucin binding efficiency of about 85%.

**Figure 3:**
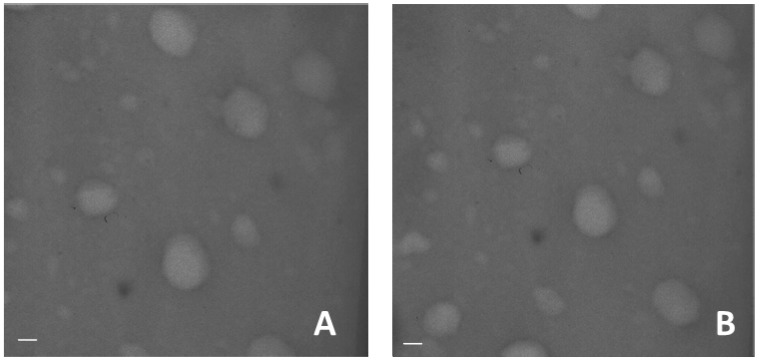
TEM image of blank (**A**) and dexamethasone-loaded nanogels (**B**) (scale bar 150 nm).

Transmission electron microscope image of blank and dexamethasone-loaded nanogels show the spherical shape of the system and confirmed the small sizes (Figure 3).

**Figure 4:**
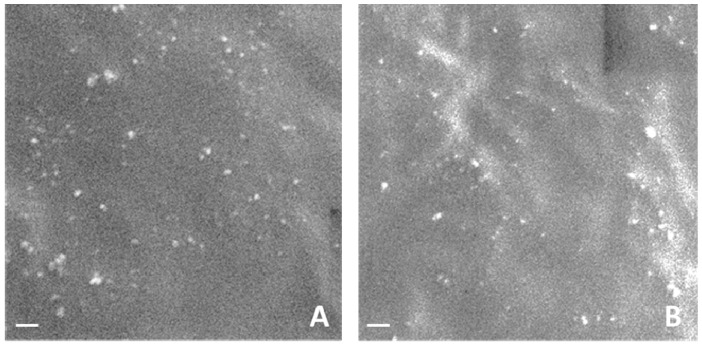
SEM image of blank (**A**) and dexamethasone-loaded nanogels (**B**) (scale bar 1 μm).

Figure 4 reports SEM images of the blank and dexamethasone-loaded nanogels.

**Figure 5:**
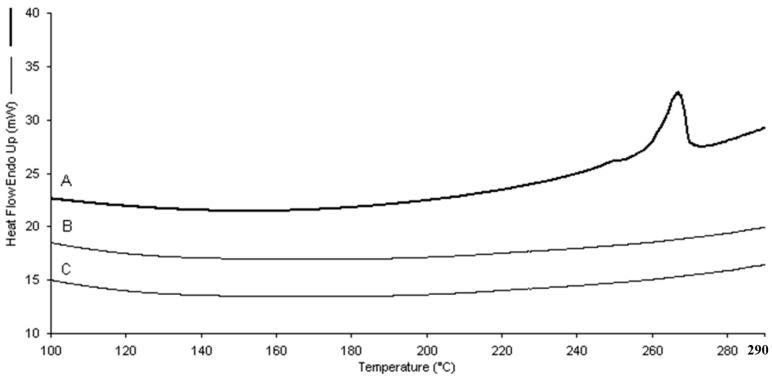
DSC thermograms of dexamethasone (**A**) and dexamethasone-loaded β-CD/PAA (**B**) and γ-CD/PAA (**C**) nanogels.

Differential scanning calorimetry (DSC) analysis showed the incorporation of dexamethasone in the two types of nanogels. The absence of the drug endothermic peak (262–264 °C) in the thermograms of the loaded nanogels confirmed the occurrence of dexamethasone molecular interactions with the polymer matrices (Figure 5).

**Figure 6:**
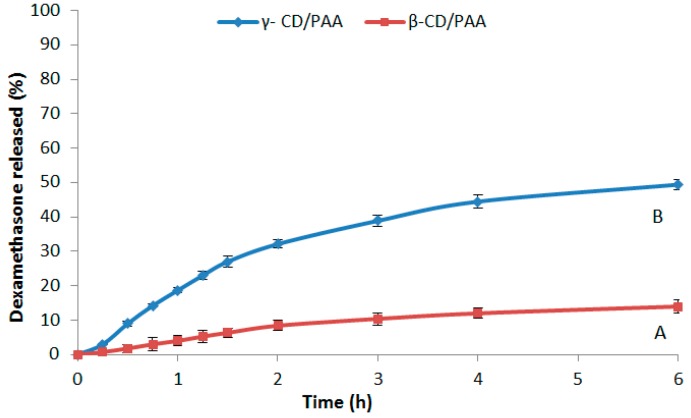
In vitro release kinetics of dexamethasone from β-CD/PAA (**A**) and γ-CD/PAA (**B**) nanogels at pH 7.4. Results are shown as means ± SEM from three independent experiments (*n =* 3).

The in vitro release kinetics of dexamethasone from β-CD/PAA and γ-CD/PAA nanogels are reported in Figure 6.

Both nanogels showed a slow and prolonged release profile over time at pH 7.4 and no initial burst effect was observed. In particular, only 14% of dexamethasone was released from the β-CD/PAA nanogel after 6 h. On the contrary, after 6 h the percentage of dexamethasone recovered in the receiving phase from γ-CD-PAA was about 50%.

Concerning nanogel safety, no significant hemolysis caused by β-CD/PAA and γ-CD/PAA nanogels, either blank or dexamethasone-loaded, was observed, confirming their good biocompatibility and the presence of tonicity values suitable for ocular administration.

Based on their smaller size values, higher loading capability and on the slower release kinetics, β-CD/PAA nanogel was selected for the cell experiments and was named as nanodexa.

### 2.2. Effect of Dexamethasone or Nanodexa on Jurkat Cell Adhesion to IL-1β-Stimulated HUVEC

**Figure 7:**
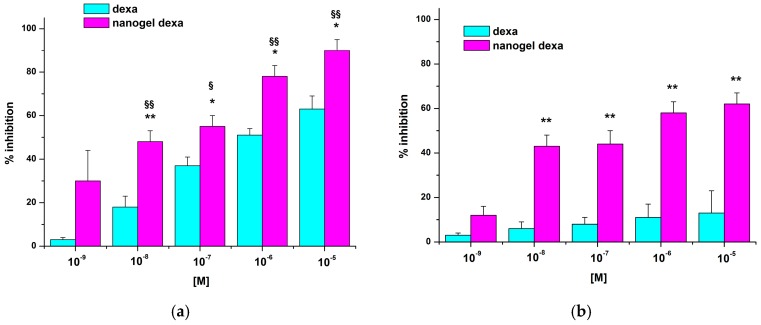
Effect of HUVEC treatment with dexa and nanodexa on adhesiveness to Jurkat cells. HUVEC were pretreated or not with titrated amounts of dexa and nanodexa (10^−9^–10^−5^ M) for 24 h (**a**) and 48 h (**b**), stimulated with IL-1β for 18 h, then incubated with Jurkat cells for 45 min. Data are expressed as mean ± SEM (*n* = 5) of the percentage of inhibition versus the control.* *p* ≤ 0.05 and ** *p* ≤ 0.01 nanodexa versus dexa (significance was assessed with Student’s *t*-test for paired varieties). ^§^ *p* < 0.05; ^§§^ *p* < 0.01, significantly different from untreated cells.

GCs act on endothelial cells by decreasing vascular permeability, adhesion molecule expression, and production of IL-1 and prostaglandins (PGs). However, Kerachian et al. [19] also showed that high doses of GCs could sensitize HUVEC to the effect of inflammatory mediators favoring their development of pro-adhesive features. Therefore, we performed our adhesion assays using HUVEC treated or not with the pro-inflammatory cytokine IL-1β to mimic pro-inflammatory conditions. HUVEC were treated for 24–48 h with increasing concentration of either dexamethasone (dexa) or nanodexa (10^−9^–10^−5^ M), then stimulated or not with IL-1β for further 18 h and, finally, incubated with Jurkat cells in the adhesion assay for 45 min. Figure 7 shows the effect of dexa or nanodexa on Jurkat cell adhesion to IL-1β-stimulated HUVEC after 24 h of treatment.

Nanodexa inhibited Jurkat cell adhesion in a concentration-dependent manner; the effect was already significant at 10^−7^ M (about 40% inhibition), with maximal inhibition (about 60%) obtained at 10^−6^–10^−5^ M. By contrast, no significant inhibition was detected with any concentration of free dexa at this time. Extending the duration of the treatment to 48 h, adhesion inhibition was detectable also for dexa, but only in the 10^−7^–10^−5^ M range of concentrations. By contrast, nanodexa was already effective at the 10^−8^ M dose and its concentration-response curve was substantially different from that of dexa (*p* ≤ 0.01).

**Figure 8:**
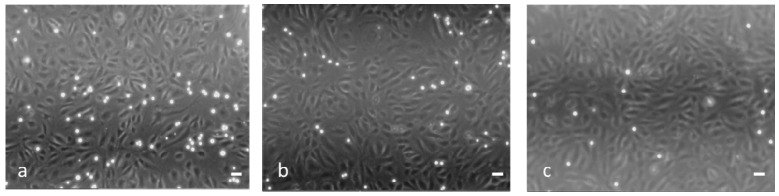
Fluorescent microscopy of Jurkat cells adherent to HUVECs that were not treated (**a**) or treated with dexa and nanodexa (**b**,**c**, respectively) (scale bar 10 μm; magnification 100×).

Figure 8 shows micrographs of the Jurkat adhesion assay on IL-1β-stimulated HUVEC untreated (a) or treated with 10^−7^ M of either dexa (b: 36% of inhibition) or nanodexa (c: 55% of inhibition) for 48 h.

### 2.3. Effect of Dexamethasone or Nanodexa on COX-2 Expression in Stimulated Jurkat Cells

**Figure 9:**
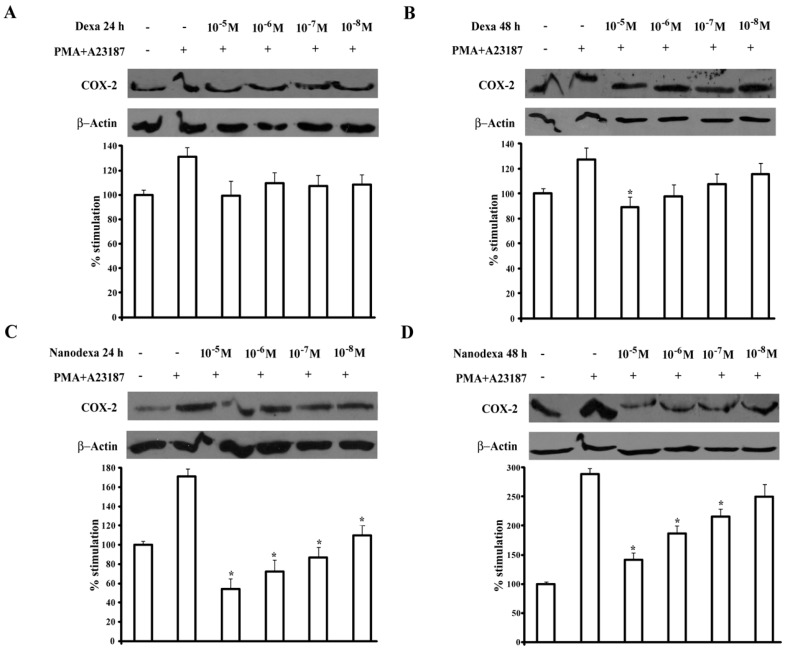
Effect of dexa or nanodexa on COX-2 expression in stimulated Jurkat. Jurkat were pretreated or not with titrated amounts of dexa or nanodexa (10^−8^–10^−5^ M) for 24 h (**A**,**C**, respectively) and 48 h (**B**,**D**, respectively) and then stimulated with PMA+A23187 for 18 h. Then, cells were lysed, and COX-2 expression was analyzed by Western blot. The bar graphs show data (mean ± SEM) normalized to β-actin, expressed as the percentage of inhibition versus the control. * *p* ≤ 0.05 (significance was assessed with one-way ANOVA and the Dunnett test). Top: Western blot analysis from a representative experiment. Bottom: Densitometric analysis of COX-2 expression expressed in arbitrary units of three independent experiments.

COX-2 is upregulated during pathological conditions, such as inflammation and cancer, and glucocorticoids inhibit induction of COX-2 expression in a variety of cell lines and in response to different stimuli [20,21]. Since COX-2 is upregulated in T cells upon activation, we compared the activity on this induction exerted by dexa and nanodexa in Jurkat cell activated by the phorbol ester PMA (15 ng/mL) and calcium ionophore A23187 (1 μM) [22] Jurkat cells were treated for 24–48 h with increasing concentration of dexa and nanodexa (10^−8^–10^−5^ M) and COX-2 expression was then stimulated with PMA+A23187. Figure 9 shows the effect of dexa and nanodexa after 24 h (panel A,C) and 48 h (panel B,D) of treatment. While dexa inhibited COX-2 expression only by 35% at 10^−7^ M after 48 h incubation, nanodexa was already maximally active after 24 h in the 10^−8^–10^−5^ M concentration range.

## 3. Discussion

Two cyclodextrin-based nanogels were obtained with attractive and promising properties as new biomaterial for dexamethasone ocular delivery. A polymer architecture comprising cross-linked cyclodextrin units was purposely tuned to load lipophilic molecules in a hydrophilic matrix. Indeed, the two nanogels were able to incorporate dexamethasone in good amounts due to the interaction with the hydrophobic cyclodextrin cavities and polymer networks. It is worth noticing that dexametasone-CD complexes have been previously described in the literature [23,24,25]. Dexamethasone has a poor water solubility (about 0.1 mg/mL) and CDs showed the capability to improve the solubility and the topical bioavailability of the drug [5]. Previous studies suggested that the A-ring of the steroid molecule was predominantly included in the cavity of CDs. The inclusion complexation of dexamethasone with a number of CDs were determined, showing that the stability constant values of β-CD and γ-CD complexes were 9560 and 37,300 M^−1^, respectively [26]. Here, the nanogel formulations were designed to increase the apparent water solubility of the drug and to enhance the contact time with the eye surface, key factors for improving the ocular drug delivery.

The β-CD/PAA nanogel provided a more stable complexation of dexamethasone than γ-CD/PAA, as the drug loading capacity and the in vitro release kinetics demonstrated. This behavior appeared in contrast with the stability constant values of the dexamethasone with the two parent CDs, as previously reported [26]. Indeed, we speculated that the bonding of CDs with the cross-linking agents in the nanogels may distort the CD cavity, so modifying the complexation capability with a guest molecule, as previously observed with alkylcarbonates derivatives of γ-cyclodextrins [27].

Intriguingly, the insertion of cyclodextrin moieties did not affect the high hydration properties of nanogels and the pH sensitive swelling capacity. Β-CD/PAA showed a greater water content in comparison to the one of γ-CD/PAA. Different cross-linking degree and dimensions of network nanochannels might be present in the polymer matrix of β-CD/PAA and γ-CD/PAA, due to the different sizes of the two cyclodextrins, i.e., 262 and 427 Å^3^ for β-CD and γ-CD, respectively. The differences in the polymer nanostructure might affect the water uptake capability of the two nanohydrogels.

The in vitro release study showed prolonged release profiles of dexamethasone without a burst effect, but the drug kinetics varied according to the type of nanogels. The observed differences might be related to the presence of different nanochannel network in the polymer matrices, besides different interactions with the CD cavities. The slow and constant dexamethasone release might be useful to achieve a sustained drug concentration in the tear fluid on the eye surface.

For effective topical drug delivery into the eye, various polymer nanodelivery systems have been described in the literature showing a great potential to control the release kinetics and the penetration of corticosteroid [9,23].

Considering a possible future clinical application of the new dexamethasone delivery system the pharmacology activity was evaluated.

Dexamethasone is widely used for its ocular anti-inflammatory effect [1,28,29,30] but can also treat lymphoid neoplasia [31], and it has been used in solid cancer therapies in combination with antitumor drugs, such as 5-fluorouracil and cisplatin [32]. It exerts its anti-inflammatory and immunosuppressive effects by reducing the expression of cytokines and adhesion molecules, inhibiting leukocyte trafficking and access to inflammation sites and interfering with leukocyte, fibroblast and endothelial cell function. In this work, to evaluate the nanodexa activity, Jurkat cells were used to assess the inhibitory effect on adhesion to human umbilical vein endothelial cells (HUVEC) and COX-2 expression. Interestingly, we found that nanodexa inhibited Jurkat cell adhesion to HUVEC and COX-2 expression with earlier effects and at lower doses than free dexamethasone.

The anti-inflammatory and immunosuppressive actions of GCs are exerted by two different mechanisms. In the classic genomic pathway, dexamethasone modulates the expression of proteins via their interactions with the cytosolic GC receptor (GR). However, GCs can also act by non-genomic mechanisms [33]. The genomic pathway is considered responsible for many adverse effects of GCs, most of which are time- and dose-dependent, such as osteoporosis, osteonecrosis, cataracts, hyperglycemia, coronary heart disease, and cognitive impairment, among others [34]. Consequently, the dose decrease is a key factor to control side effects of dexamethasone.

The marked anti-inflammatory effect obtained with nanodexa at lower concentration of the drug may play an important role in decreasing the administered dose and the adverse side effects.

Inflammation involves adhesive interactions between circulating leukocytes and endothelial cells lining the vascular wall. In response to various stimuli, such as the pro-inflammatory cytokines TNF-alfa, IL-1 beta, INF-gamma, and endothelial cells undergo inflammatory activation, producing an increased surface expression of cell adhesion molecules (CAMs), such as ICAM-1, VCAM-1, and E-selectin [35]. These endothelial CAMs play a fundamental role in leukocyte recruitment from the blood for tissue infiltration. Chronic induction of these CAMs leads to abnormal leukocyte recruitment, as seen in chronic inflammatory diseases [36]. Therefore, the effective inhibitory activity displayed by nanodexa on CAM expression by “inflamed” endothelial cells may be beneficial in blunting detrimental inflammatory reactions [37,38].

Even the potent inhibitory effect of nanodexa on COX-2 expression is intriguing. Prostaglandins (PGs) are known to be an important mediators of acute inflammation. They are synthesized by cyclooxygenase, comprising the constitutively expressed isoform COX-1 and the inducible isoform COX-2. COX-1 is expressed in most tissues that generate PGs during their normal physiological functions, and its expression does not fluctuate in response to stimuli. In contrast, COX-2 induction has critical roles in the response to tissue injury and infection and is an essential component of the inflammatory response and tissue repair [39]. Although the physiological activity of COX-2 may provide a definite benefit to the organism, its aberrant or excessive expression has been implicated in the pathogenesis of many diseases, such as chronic inflammations [40]. Taking into account that the positive effects of dexamethasone-loaded nanogels are exerted through earlier effectiveness and at lower doses than those of free dexamethasone, it may be hypothesized that this innovative nanogel formulation may increase the therapeutic efficacy with less adverse reactions. Finally, the small sizes and the mucoadhesive property of nanodexa can favor the ocular retention of the nanoformulation, contributing to the sustained release of the drug at the target site [41]. This might provide a long-lasting pharmacological activity with less frequent dexamethasone instillations.

## 4. Conclusions

Two promising cyclodextrin-based pH-sensitive nanogels were obtained. Morphological and physico-chemical properties of the dexamethasone-loaded nanogels made them suitable as prolonged release delivery systems for ocular administration.

Taken together, the in vitro results demonstrated that the β-CD/PAA dexamethasone nanoformulation is effective at lower doses and with faster onset compared to the free drug.

Therefore, dexamethasone in CD-PAA nanogels may represent a potential novel strategy to formulate eye drops able to overcome the shortcomings of this drug.

## 5. Materials and Methods

### 5.1. Materials

All materials were from Sigma-Aldrich (St. Louis, MO, USA). All reagents were of analytical grade.

### 5.2. PAA Hydrogel Synthesis

Two hydrogels were prepared as previously described [16] with modifications. Briefly, the polyaddition reaction between *prim*-or bis-*sec*-amines and bisacrylamides leads to polyamidoamines (PAAs). At pH ≥ 11.5 the hydroxyl groups of cyclodextrins react with bisacrylamides in the same way and cyclodextrins behave as multifunctional monomers and give cross-linked polymer named nanosponges. With bisacrylamides, mixtures of cyclodextrins and amines give copolymeric hydrogels. The CD-containing hydrogels used in this work were prepared from 2,2-bisacrylamido acetic acid (BAC) as bisacrylamide, 2-methyl piperazine (2-MP) as amine and β- or γ-cyclodextrin. The resultant hydrogels were named β-CD/PAA and γ-CD/PAA, respectively. In them, the PAA short chains connecting the CD moieties contained carboxyl- and amine groups and, in principle, had amphoteric properties. In a typical procedure, β-CD/PAA was prepared dissolving β-CD (734.84 mg, 0.62 mmol) and LiOH·H_2_O (77.7 g, 1.83 mmol) in H_2_O (0.4 mL) in a test tube. BAC (188.7 mg, 0.935 mmol) and LiOH·H_2_O (39.6 mg, 0.935 mmol) were dissolved in H_2_O (0.3 mL) in a second test tube, then 2-methylpiperazine (93.7 mg, 0.935 mmol) was added. The two solutions were thoroughly mixed and allowed to react at 25 °C for 48 h. The final product appeared as a homogeneous, transparent, and soft gel, which was tritured under water, and purified by repeated water/ethanol extraction cycles. The nanosponge sample was first soaked in deionized H_2_O (50 mL) and allowed swelling for 2 h, the pH was lowered to 5 with HCl 37% *w*/*w* then the nanosponge was soaked in ethanol (50 mL) for 2 h. This procedure was repeated three times. The product was dried to a constant weight. Yield: 76%.

γ-CD/PAA was prepared following the same procedure described for β-CD/PAA. The monomers used and their amounts are reported below. The γ-CD was dissolved at about 95 °C.

γ-CD/PAA: γ-CD (1030.52 mg, 0.77 mmol), LiOH·H_2_O (158.4 mg, 3.74 mmol), H_2_O (0.7 mL), BAC (192.4 mg, 0.96 mmol), LiOH·H_2_O (40.8 mg, 0.96 mmol), H_2_O (0.3 mL), 2-methylpiperazine (96.3 mg, 0.96 mmol). Yield: 45%.

### 5.3. FTIR Analysis

Fourier transformed infrared (FTIR) spectra of β-CD and β-CD/PAA were obtained using a Perkin Elmer Spectrum 100 FT-IR in the region of 4000–650 cm^−1^. Data acquisition was done by Spectrum software version 10.03.05 (Perkin Elmer Corporation, Waltham, MA, USA).

### 5.4. Swelling Capacity Evaluation

The swelling capacity of nanogels was evaluated by gravimetric analysis. A weighted amount of dry β-CD/PAA or γ-CD/PAA (*W*_d_) was dispersed in 5.0 mL of PBS buffer at different pH values (i.e., 1, 4, 5, 6, 7.4). The mixture was stirred at 25 and 37 °C overnight. The supernatant was removed and then the wet weight (*W*_w_) of the nanogels was measured. The swelling capacity (S_c_) was calculated as the following equation: *S*_c_ = (*W*_w_ − *W*_d_)/*W*_d_.

### 5.5. Nanogel Preparation by HPH

Nanogels were obtained using a top down method. To this purpose, β-CD/PAA or γ-CD/PAA were suspended in saline solution (NaCl 0.9% *w*/*v*) at a concentration of 10 mg/mL and homogenized using a high shear homogenizer (Ultraturrax) for 10 min at 24,000 rpm. To further reduce the size of the nanogels and obtain an almost homogenous nanoparticle distribution, the sample underwent to high pressure homogenization for 90 min at a back-pressure of 500 bar, using an EmulsiFlex C5 instrument (Avastin, Ottawa, ON, Canada). The nanogels were then purified by dialysis (membrane cutoff of 12,000 Da) to eliminate potential synthesis residues.

### 5.6. Preparation of Dexamethasone-Loaded Nanogel

Dexamethasone was incorporated in the pre-formed nanogels, by the addition of dexamethasone (1 mg/mL) to the aqueous nanosuspension of β-CD/PAA or γ-CD/PAA. Then, the mixture was stirred at room temperature in dark conditions over night. A purification step by dialysis (Spectrapore, Rancho Dominguez, CA, USA, cellulose membrane, cutoff of 12,000 Da) was carried out to eliminate the unbounded drug.

### 5.7. Physico-Chemical Characterization of Nanogels

The average diameters and polydispersity indices of nanogels were determined, after their dispersion in water, by photon correlation spectroscopy (PCS) using a 90 Plus Instrument (Brookhaven, NY, USA) at a fixed angle of 90° and at a temperature of 25 °C. Each sample was analyzed in triplicate. The nanogel zeta potentials were measured by electrophoretic mobility (90 Plus Instrument, Brookhaven, NY, USA). For zeta potential determination, the samples were diluted in water and placed in the electrophoretic cell, where an electric field of approximately 15 V/cm was applied. The morphology of nanogels was evaluated by scanning electron microscopy and transmission electron microscopy. For TEM analysis a Philips CM 10 transmission electron microscope was used. The samples were sprayed on Formwar-coated copper and air-dried before observation.

### 5.8. Thermal Analysis

Differential scanning calorimetry (DSC) was carried out by means of a Perkin Elmer DSC/7 differential scanning calorimeter (Perkin-Elmer, Waltham, MA, USA) equipped with a TAC 7/DX instrument controller. The instrument was calibrated with indium for melting point and heat of fusion. A heating rate of 10 °C/min was employed in the 25–250 °C temperature range. Standard aluminum sample pans (Perkin-Elmer) were used; an empty pan was used as a reference standard. Analyses were performed in triplicate on 3 mg freeze-dried samples under a nitrogen purge.

### 5.9. In Vitro Release Kinetics of Dexamethasone from Nanogels

In vitro drug release experiments were conducted in a multi-compartment rotating cell, comprising a donor chamber separated by a cellulose membrane (Spectrapore, cut-off = 12,000 Da) from a receiving chamber. One milliliter of dexamethasone-loaded nanogels was placed in the donor chamber. The receiving compartment contained 1 mL of phosphate-buffered saline (PBS) at pH 7.4 with 0.1% sodium dodecyl sulfate (SDS) to assure drug solubility. The receiving phase was withdrawn at regular intervals and completely replaced with the same amount of fresh solution, to maintain sink conditions. The concentration of dexamethasone in the withdrawn samples was detected by HPLC.

For the dexamethasone quantitative determination an HPLC analysis was performed using a Perkin Elmer pump (Perkin Elmer PUMP 250B, Waltham, MA, USA) equipped with a spectrophotometer detector (Flexar UV/Vis LC spectrophotometer detector, Perkin Elmer, Waltham, MA, USA). A reversed phase Agilent TC C18 column (150 cm × 4.6 mm, pore size 5 μm; Agilent Technologies, Santa Clara, CA, USA) was used. A mixture of acetonitrile −25 mM phosphate buffer pH 3 (27:73, *v*/*v*) was used as the mobile phase at a flow rate of 1.0 mL/min and the effluent monitored by measuring absorbance at 246 nm. HPLC chromatograms of dexamethasone are reported in Supplementary Material (Figure S1).

### 5.10. Mucoadhesion Test

The in vitro evaluation of the mucoadhesive properties of nanogels was carried out. To this purpose, the interaction between mucin and nanogels was determined by turbidimetric assay.

Stock solutions of mucin (from porcine stomach) were prepared in water in the concentration range from 0.1 to 1 mg/mL. The transmittance of mucin solutions was measured at 500 nm with an UV spectrophotometer and a calibration curve was obtained. 1 mL of β-CD/PAA suspension was mixed with mucin stock solution and incubated under magnetic stirring for 30 min. Then, the samples were centrifuged for 5 min at 10,000 rpm and the transmittance of the supernatant, which contains the mucin non-adhesive to the β-CD/PAA hydrogel, was measured at 500 nm.

### 5.11. Determination of the Hemolytic Activity

For hemolytic activity determination, 100 μL of nanogels were incubated at 37 °C for 90 min with diluted blood (1:4 *v*/*v*) obtained by adding freshly-prepared PBS at pH = 7.4. After incubation, nanogels-containing blood was centrifuged at 1000 rpm for 5 min to separate plasma. The amount of hemoglobin released due to hemolysis was determined spectrophotometrically (absorbance readout at 543 nm using a Duo spectrophotometer, Beckman, Brea, CA, USA). The hemolytic activity was calculated to reference with nanogel-free diluted blood. Complete hemolysis was induced by the addition of ammonium sulfate (20% *w*/*v*).

Optical microscopy was used to evaluate changes in red blood cell morphology after incubation with the formulations.

### 5.12. Cell Culture

HUVEC were isolated from human umbilical veins by trypsin treatment (1%) and cultured at 37 °C (5% CO_2_, 85–95% humidity) in M199 medium with the addition of 20% fetal calf serum (FCS) and 100 U/mL penicillin, 100 μg/mL streptomycin, 5 UI/mL heparin, 12 μg/mL bovine brain extract, and 200 mM glutamine. HUVEC were grown to confluence in flasks and used at the 2nd–5th passage. Informed consent was obtained from all donors. All subjects gave their informed consent for inclusion before they participated in the study. The study was conducted in accordance with the Declaration of Helsinki, and the protocol was approved by the Ethics Committee of Turin University (Project DIAC_RLO1601). HUVEC viability was not affected by the treatment with the drug.

Leukemic human T cells (Jurkat, clone E6-1) were obtained from the American Type Culture Collection (ATCC; Manassas, VA, USA), and were cultured in RPMI 1640 medium supplemented with 10% FCS, 100 U/mL penicillin, and 100 μg/mL streptomycin at 37 °C in a 5% CO_2_ humidified atmosphere.

### 5.13. Cells Adhesion Assay

HUVEC were grown to confluence in 24-well plates, washed, and rested for one day in M199 plus 10% FCS. The cells were incubated or otherwise with increasing concentrations of dexa or nanodexa (10^−9^–10^−5^ M) for 24–48 h, washed with fresh medium twice, stimulated for 24 h with IL-1β 1 ng/mL, and incubated with the Jurkat cells (1 × 10^5^ cell/well) for 45 min; this incubation time was chosen to allow full sedimentation of the adhering cells. After incubation, non-adherent cells were removed by being washed three times with M199. The centre of each well was analysed by fluorescence image analysis [33]. Adherent cells were counted by the Image Pro Plus Software for micro-imaging (version 5.0, Media Cybernetics, Rockville, MD, USA). Single experimental points were assayed in triplicate, and the standard error of the three replicates was always below 10%. Percentage inhibition of adhesion was calculated as follows: (100 − (*a*)/(*b*)) × 100, where *a* is adhesion measured in the presence of the compound plus stimulus minus basal adhesion and *b* is adhesion elicited by stimulus minus basal adhesion. The adhesion measured on untreated cells was 25 ± 2 cells/microscope fields, and on IL-1β-stimulated cells was 58 ± 6 cells/microscope fields (*n* = 19).

### 5.14. Protein Extraction and Western Blot Analysis

In order to compare dexa with nanodexa effects on COX-2 expression, Jurkat cells were pre-treated with increasing concentrations of the drugs (10^−8^–10^−5^ M) for 24–48 h, and then stimulated with Phorbol 12-myristate 13-acetate (PMA) 15 ng/mL + A23187 1 µM for 18 h.

Cells were lysed in a buffer composed of 50 mM Tris-HCl pH 7.4, 150 mM NaCl, 5 mM EDTA, 1% NP40, phosphatase, and protease inhibitor cocktails. Cell lysates were cleared from insoluble fractions through high-speed centrifugation, and protein concentrations were determined with a commercially available kit (Bio Rad Laboratories, Hercules, CA, USA). Then, 10–40 µg proteins were separated on 10% SDS PAGE gels and transferred onto nitrocellulose membranes. These were blocked by incubation for 1 h at room temperature with 5% nonfat milk dissolved in TBS Tween 20. The membranes were probed overnight with the primary antibodies (Cell Signaling, Danvers, MA, USA, dilution 1/1000) and, after three washes, incubated for 1 h with horseradish peroxidase (HRP)-conjugated secondary antibodies. Bands were detected by chemiluminescence, and densitometric analysis was performed with the Multi-Analyst software (version 1.1, Bio-Rad Laboratories, Hercules, CA, USA).

### 5.15. Statistical Analysis

If not differently stated, data are expressed as means ± SEM. (*n* = 3). Statistical analysis was performed with GraphPad Prism 4.0 software (Graphpad Software Inc, San Diego, CA, USA). Significance was assessed with Student’s *t*-test for paired varieties or with the one-way ANOVA and the Dunnett test with *p* ≤ 0.05 as the cut-off.

## Figures and Tables

**Table 1:** Sizes and ζ potential values of dexamethasone-loaded β-CD/PAA and γ-CD/PAA nanogels.

Loaded Nanogels	Average Diameter (nm)	Polydispersity Index	ζ-Potential (mV)
β-CD/PAA	314 ± 3.5	0.10	−29.85 ± 1.5
γ-CD/PAA	372 ± 5.1	0.11	−33.30 ± 1.8

## Supplementary Materials

The following are available online at www.mdpi.com/2310-2861/3/2/22/s1.

## Abbreviations

The following abbreviations are used in this manuscript:

HUVEC	human umbilical vein endothelial cells
Polyamidoamines	(PAAs)
Cyclodextrins	(CD)
2,2-bisacrylamidoacetic acid	(BAC)
2-methylpiperazine	(2-MeP)
High Pressure Homogenization	(HPH)
Glucocorticoids	(GCs)
Phorbol 12-myristate 13-acetate	(PMA)
GC receptor	(GR)
Prostaglandins	(PGs)
