# Supplementary material for: Cyclodextrin-Based Nanohydrogels Containing Polyamidoamine Units: A New Dexamethasone Delivery System for Inflammatory Diseases
Source: Gels. 2017 Jun 8;3(2):22. doi: 10.3390/gels3020022 (PMC6318607; doi:10.3390/gels3020022)
Supplement: Supplementary file 1 [file gels-03-00022-s001.pdf]

Cyclodextrin-Based Nanohydrogels Containing Polyamidoamine Units: A New Dexamethasone Delivery System for Inflammatory Diseases

Monica Argenziano, Chiara Dianzani, Benedetta Ferrara, Shankar Swaminathan, Amedea Manfredi, Elisabetta Ranucci, Roberta Cavalli and Paolo Ferruti

A

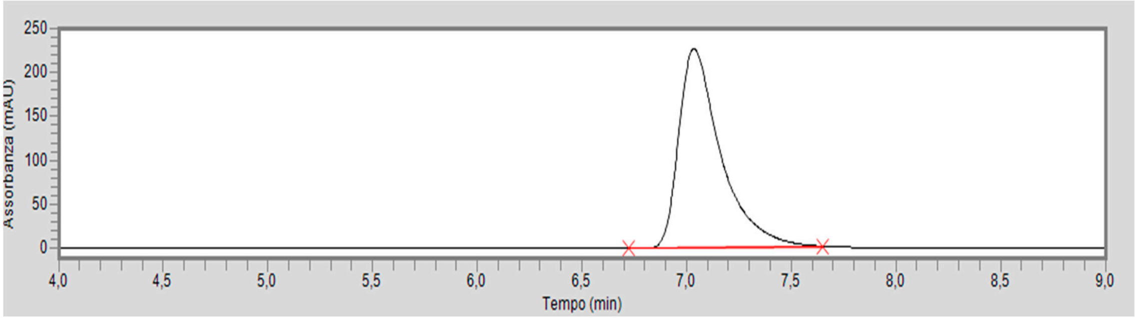

B

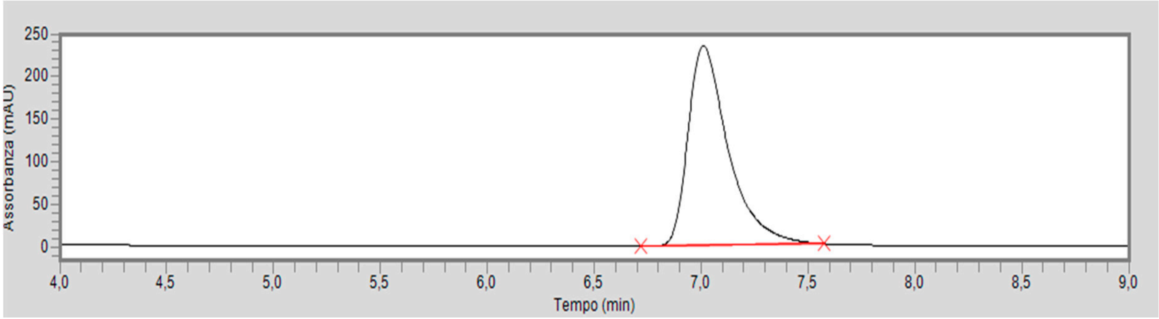

Figure S1. Chromatograms of dexamethasone standard solution (**A**) and dexamethasone released from β-CD/PAA (**B**).
